# Applying the Principles of Trauma-Informed Care to the Evaluation and Management of Patients Who Undergo Metabolic and Bariatric Surgery

**DOI:** 10.1007/s11695-024-07597-4

**Published:** 2024-11-27

**Authors:** David B. Sarwer, Krista Schroeder, Sarah R. Fischbach, Sophia M. Atwood, Leslie J. Heinberg

**Affiliations:** 1 Center for Obesity Research and Education, College of Public Health, Temple University, Suite 175, 3223 N. Broad St, Philadelphia, PA 19104 USA; 2 Department of Nursing, Temple University College of Public Health, Philadelphia, PA USA; 3 Department of Surgery, Cleveland Clinic Lerner College of Medicine, Cleveland, OH USA

**Keywords:** ACEs, Adverse childhood experiences, Metabolic and bariatric surgery, Obesity, Trauma, Trauma-informed care

## Abstract

Evaluation of relevant psychosocial variables is an important aspect of comprehensive, high-quality metabolic and bariatric surgery (MBS) care. Given the high rates of adverse childhood experience (ACEs) and other forms of trauma experienced later in life reported by individuals with class III obesity, it is time to apply the principles of trauma-informed care to the multidisciplinary care of MBS patients. This narrative review begins with a summary of the literature on the psychosocial functioning of individuals who present for MBS. Emphasis is placed upon the relationship between ACEs, class III obesity, and MBS. Trauma-informed care is defined, and its principles are applied to the MBS care continuum. The paper ends with a recommendation on how the field of MBS can integrate trauma-informed care into clinical practice and future research.

## Introduction

Individuals with more severe forms of obesity often present for treatment while experiencing significant psychosocial burden [1–3]. Patients presenting for metabolic and bariatric surgery (MBS) often report low self-esteem, body image dissatisfaction, and impaired quality of life. Many have symptoms of one or more psychiatric diagnoses, including mood disorders, anxiety, substance use disorder, and disordered eating [1]. Some work in this area has suggested that the rates of these untoward psychological consequences are higher than seen in persons with obesity who do not seek treatment. While this is an important research question, this paper will focus on what is known about those individuals who seek and undergo MBS.

Following MBS, the majority of patients experience improvements psychosocial status and functioning. Within the first postoperative year, patients report improvements in self-esteem, body image, and both health- and weight-related quality of life, as well as decreased mental healthcare utilization [4]. However, a substantial minority experience significant mental health issues. For example, there is an increase in alcohol use disorders, notably during the second postoperative year [1, 5–8]. The rate increases to approximately 20% of individuals by the sixth postoperative year [7, 8]. Studies also have shown an increased risk of self-injurious behaviors and suicidality amongst MBS patients compared to others with a similar body mass [1, 9].

Much of the work on the psychosocial aspects of MBS has focused on individual level variables. However, the interplay of psychosocial functioning and medical care does not occur in a vacuum; it is the product of one’s past and current lived experiences and environment. Consideration of these issues in relationship to MBS is consistent with an extensive body of theory that recognizes the influence of multiple levels of ecology on health outcomes [10]. It also is consistent with research that has documented the effects of such factors on both psychosocial well-being and body weight [11–19].

These relationships have been largely overlooked in the MBS literature. Recognizing the complexities in the physical, social, behavioral, psychological, and environmental makeup of individuals with obesity presenting for MBS can encourage optimally comprehensive care. It also would support the delivery of patient-centered care, when the lived experience of the patient, both before and after surgery, is considered by all members of the treatment team [20–23].

A patient’s history of adverse childhood experiences (ACEs) can play an important role in psychosocial functioning and well-being throughout the MBS process. ACEs are defined as traumatic experiences that occur during childhood, including physical, psychological, or sexual abuse; neglect; family members’ substance abuse, mental illness, or criminal behavior; interpersonal violence against a caregiver; and parent separation or divorce [24]. Of note, ACEs are a subset of trauma (limited to events that occur during childhood) that can have a particularly salient impact given the effects of trauma on a child’s developing neuroendocrine and physiological functioning, as well as how childhood trauma can impact how a child interacts with and perceives their world for the rest of their life [25]. Given that ACEs are one of the most widespread causes of trauma, appreciating the relationship of ACEs and obesity allows for the consideration of the use of principles of trauma-informed care to the comprehensive pre- and postoperative care of MBS patients. However, the principles of trauma-informed care will be beneficial to patients independent of when traumatic experiences have occurred.

Trauma-informed care is a well-established approach to service and care delivery that recognizes and responds to the impacts of trauma. It also actively avoids re-traumatizing individuals. Recently, it has been considered and/or actively applied related to other forms of medical care, educational settings, and neighborhoods [26–28]. Given the number of individuals who present for MBS with a history of ACEs, as well as the potential for re-traumatization during pre-, peri-, or postoperative care, applying principles of trauma-informed care to bariatric surgery is an overdue evolution in the high-quality, multidisciplinary treatment of severe obesity.

Despite the effect of high prevalence of psychosocial burden and ACEs among populations seeking MBS, preoperative psychosocial evaluation of patients is not the global standard of care. Even when these evaluations are performed, it is unclear how regularly ACEs are assessed. It is perhaps as likely, if not more so, that non-mental health professionals may become aware of a patient’s ACEs history and, as a result, need to be prepared to address the issue thoughtfully and appropriately. Further, we believed the application of the principles of trauma informed care is highly novel and has received little, if any, attention in the MBS academic literature and is an important and clinically relevant issue for the entire MBS treatment team.

With this context in mind, this narrative review begins with a brief summary of literature on the psychosocial functioning of individuals who present for MBS. Following, the paper moves to a detailed discussion of the relationship between ACEs, class III obesity, and MBS. Trauma-informed care is then defined and its principles applied to the MBS care continuum.

## Psychosocial Functioning of Individuals Who Present for MBS

As noted above, many individuals seeking MBS present to the treatment team with impaired psychosocial functioning. Up to 30% of bariatric patients report current depression, with up to 70% reporting at least one episode of depression in their lifetime [1, 29–31]. Up to 24% of patients present with a current anxiety disorder and 38% present with a lifetime diagnosis [32]. Eating disorders are also common; fifty percent of patients experience some form of disordered eating, and binge eating disorder is diagnosed in 15% of patients [1, 33]. Though less than 2% of patients present with a current substance use disorder (SUD) at the time of surgery, history of SUDs have been reported in up to 33% of patients [1, 34]. Some of these studies have made comparisons between those who seek and undergo MBS; others have not. The consensus, as seen in other areas of the obesity treatment literature, is that rates of these conditions are often higher among those who are seeking treatment than those with similar severity of obesity in the general population.

## ACEs, Obesity, and MBS

The physiological and psychological outcomes of ACEs may play a crucial role in the development of obesity [35–39]. The ACE-obesity association is hypothesized to arise from a diverse range of pathways that connect the lifespan effects of childhood ACEs to elevation in body adiposity across the lifespan. For example, ACE-associated stress may lead to hypothalamic pituitary access dysregulation, changes in insulin and hunger hormone levels, cytokine activation, and immunometabolic derangements that increase obesity risk [40–43]. Childhood trauma has been linked both directly and indirectly to obesity via elevated levels of C-reactive protein, which leads to inflammation. The stress also promotes secretion of glucocorticoids, hormones that at elevated levels cause weight gain and, in turn, obesity [37].

Mental health conditions associated with ACE exposure, including depression and binge eating disorder, are well-established risk factors for obesity [35, 44–46]. Depression, self-criticism, dissociation, post-traumatic stress disorder (PTSD) symptoms, and eating disorder symptoms have been shown to moderate the relationship between early life adversity and obesity [27]. ACE-associated stress also is related to unhealthy coping behaviors, such as eating highly palatable, calorically-dense foods [44, 47, 48]. Household dysfunction connected to ACEs may detract from developing routines and behaviors associated with maintaining a healthy weight [35]. While research findings are not entirely consistent, certain types and/or frequency of ACEs — such as sexual abuse among women and higher number of ACEs experienced — are associated with higher risk for the development of obesity [26, 49, 50].

Given the relationship between ACEs and obesity, it is not surprising that ACEs are common in patients presenting for MBS [38]. National estimates suggest the 60% of American adults have experienced an ACE [36, 51, 52]. Among individuals presenting for MBS, rates of childhood maltreatment are as high as 67% in females and 47% in males, with 24% reporting 4 or more ACEs [52–54]. Those who experience childhood maltreatment (one type of ACE) often present with higher rates of physical inactivity, type II diabetes, smoking behaviors, heavy alcohol use, substance use disorder, and psychiatric disorders [37, 54]. Additionally, childhood maltreatment is associated with psychological distress, including depression and PTSD [37, 54, 55]. These effects of ACEs can influence patients’ engagement, outcomes, and experiences with MBS. ACEs’ long-term effects on coping with stress, emotion management, and interpersonal trust may also influence the potential for a patient to appropriately partner with the clinical team and adhere to post-MBS dietary and behavioral recommendations.

A number of studies have investigated the relationship between ACEs and postoperative weight loss [34, 56–60]. This literature is largely inconclusive—some studies have identified an association between a history of ACEs and postoperative weight loss while others have not. How ACEs were operationalized or assessed, as well as other methodological differences across the studies, likely account for the lack of conclusiveness with respect to the relationship. Beyond weight loss, it appears that MBS patients with a history of ACEs are exposed to a range of untoward postoperative psychological and physical experiences. Patients with a history of ACEs demonstrate greater postoperative depression, suicidal ideation, PTSD symptoms, self-harm behaviors, and other mental health disorders [9, 38, 54, 61]. Patients with high ACE scores also have worse biomarkers of cardiovascular disease, higher total cholesterol before and after surgery, and higher postoperative BMI than those with low ACE scores [57]. Improved identification and management of this sub-group of MBS patients would likely help optimize their patient experiences and postoperative psychosocial outcomes.

## Application of Principles of Trauma-Informed Care to MBS

Given the rate of ACEs among individuals with obesity, and particularly those who present for MBS, principles of trauma-informed care should be included in multidisciplinary care of MBS patients. Trauma-informed care emphasizes the principles of safety, trustworthiness, transparency, peer support, and empowerment in its focus on delivering care in a manner that promotes healing and avoids re-traumatization [28]. Underpinning this approach is the notion that trauma has lasting consequences for physical and mental health, but that healing, flourishing, and post-traumatic growth after trauma are fully possible, particularly when individuals who experienced trauma have appropriate resources and supports.

There are many models of trauma-informed care that clinical teams can use to deliver patient-centric care; each focuses on understanding trauma, emphasizing compassion, collaboration, and empowerment, as well as building positive, healthy attachments to individuals and their environments [27]. Resources for implementing trauma-informed approaches are available from relevant non-profit organizations and expert work groups [62–65]. Collectively, they share a multi-level and systems wide approach—trauma-informed care is not solely about how an individual clinician interacts with a patient but about how a system, from the MBS program to the entire hospital or health system, is structured to support trauma-informed care delivery. It necessitates knowledge sharing between all parties involved, as well as ongoing staff education and training, appropriate policies and procedures, and environmental modification to ensure clinical atmospheres are supportive. Given the breadth of approach required to ensure its comprehensive delivery, buy-in from health system or hospital leadership and attention to sustainability for trauma-informed efforts are key for implementation and sustained success [66]. This may be challenging for financial or logistical reasons but should not be dismissed.

Understanding trauma is crucial in determining the best approach to comprehensive, empathic MBS care. In the USA, a preoperative evaluation by a behavioral health professional is required by most insurance companies, if not MBS programs. In other parts of the world, behavioral health professionals are asked to consult with patients when there is concern about psychosocial status and functioning. Such professionals are ideally suited to inquire about a history of ACEs, their relationship to the development of obesity, and their impact on current psychosocial functioning. Some, but not all, guidelines have recommended that ACEs be routinely assessed. Additionally, others argue that trauma-informed care should be delivered to all patients — regardless of ACE or adult trauma history — as a “universal precaution” given the widespread prevalence of ACEs as well as the observation that ACE screenings are unhelpful and potentially harmful if not delivered by trained professionals and without appropriate long-term resources being available [67, 68]. For teams considering ACE screening, guidance exists for integrating ACE screening into clinical workflows in a trauma-informed manner [69].

We believe that assessing ACE history in a compassionate, supportive manner is consistent with the principles of trauma-informed care. This can be done by clinical interview or psychometrically validated patient-reported outcome measures [64, 70]. Sharing of ACEs in the medical record should be done thoughtfully and with consideration that some caregivers throughout the health care system could unintentionally misuse information, as should be done with all mental health issues.

Literature about how patients with a history of ACEs experience the MBS process is lacking. However, the broader literature about trauma’s effects on health care delivery can inform an understanding of how aspects of the MBS process might be distressing or even retraumatizing for some. For example, being asked to physically undress for examinations, being seen less than fully clothed by others, and physical touch that occurs as part of the clinical exam or during procedures (e.g., ultrasounds) can be anxiety provoking.

The peri-operative period may be particularly distressing or traumatizing for patients. The anticipatory anxiety experienced by almost all surgery patients may be heightened due to lack of patient autonomy inherent to peri-operative processes, as well as the potential stressors associated with the immediate peri-operative period (e.g., transient postoperative delirium, pain). Presence of invasive equipment, such as IVs and urinary catheters, and lack of privacy in peri-operative spaces (e.g., no private toilet facilities, curtains instead of doors separating patients from one another) might also be distressing for patients who have experienced trauma. Application of the mask for anesthesia or perioperative oxygenation may produce anxiety or panic in some patients.

The physical spaces that patients experience might be challenging or traumatizing as well. MBS clinical spaces can be designed to promote feelings of safety and security, such as by reducing ambient sounds and light intensities, using natural lighting when possible, and ensuring appropriate levels of privacy [28, 71]. While most patients who undergo MBS require only a brief inpatient stay, it is important to consider how that immediate postoperative hospitalization may also lead to distress or re-traumatization. For example, hospital policies to support flexible visitation and postoperative access to staff members who offer psychosocial support (e.g., pastoral care) may be of benefit to patients. These types of environmental and policy changes — while potentially challenging given the need for financial and logistical consideration — merit consideration to ensure a truly trauma-informed MBS care delivery system.

While patients are experiencing their rapid weight loss in the first few months after surgery, most report significant improvements in psychosocial functioning. Patients typically report improvements in self-esteem, depressive symptoms, quality of life, body image, and sexual functioning [72]. These benefits may not be fully realized for those with a history of ACEs. For example, a person with a history of childhood sexual abuse may be psychologically uncomfortable if someone compliments their postoperative appearance. Similarly, while a sexual partner may be interested in increasing the frequency of sexual behavior after weight loss, the patient with a history of ACEs may not. Postoperative changes in body image and sexuality should be discussed during behavioral health visits and also can be a fruitful topic for postoperative support groups.

Given that trauma-informed approaches have not been widely examined related to MBS as of yet, work is needed to elucidate if and how trauma-informed care can be applied in this setting. That work can be guided by the consideration of how each tenant of trauma-informed care may be applied in relation to MBS. For example, the peer support tenant of MBS could be operationalized via inclusion of community health workers as part of pre-/post-MBS care. Collaboration and mutuality could be operationalized by ensuring that weight management education supports the power of the patient to participate in setting and obtaining select postoperative goals. Trustworthiness and transparency could be fostered by providing patients with clear and reliable methods of contacting clinical staff as needed and ensuring contacts are replied to in a reasonable time frame, as well as not overpromising potential beneficial effects of MBS; Safety can be underscored by ensuring clinical rooms support feelings of physical safety (e.g., participant has option to have view of doorways, minimize potential for loud startling noises such as doors slamming, appropriate ambient light), and being clear with patients about how their ACEs screening data will be protected. Patients can feel empowered by incorporating formal, structured methods for assessing patient feedback related to trauma-informed strategies into the patient feedback process. Finally, attention to cultural, historic, and gender issues could be operationalized by ensuring equity and bias training for all staff.

The effectiveness of such strategies to support trauma-informed bariatric care is likely to be care enhancing, but also can be seen as an empirical question. That work would require collaborations between diverse members of the clinical team, trauma experts, and patients who have experienced MBS. Importantly, efforts to test and implement trauma-informed bariatric processes must be developed in sensitivity to clinicians’ competing demands and time constraints to ensure feasibility, sustainability, and staff buy-in. For guidance in doing so, MBS teams can review the literature on how trauma-informed approaches have been implemented in other healthcare settings such as emergency departments and behavioral health services [73, 74].

## Future Directions

As the continuum of care for those undergoing MBS continues to evolve, consideration of the impact of ACEs on the patient’s experience is overdue. Inclusion of trauma-informed principles to MBS care is a logical strategy to ensure that the entire team is prepared to care for the large percentage of MBS patients with a history of ACEs or trauma experienced in adulthood.

Looking forward, we propose greater discussion of these issues at local, regional, national, and international meetings. For many of the disciplines represented on MBS teams, the appeal of this approach will be quite intuitive. Many team members have likely witnessed, either knowingly or unknowingly, how a history of trauma has influenced a clinical encounter. Trauma-informed care is not currently a foundational element of graduate education for most, if not all, clinical disciplines. Thus, appreciation of its importance and development of skills to identify, assess, and manage a patient with a history of trauma needs to be learned during clinical practice, in much the same way that most members of the multidisciplinary care team learned about the specifics of obesity and bariatric care. Future research should develop, test, and evaluate approaches to incorporating trauma-informed care into MBS. This is particularly true because trauma-informed care practices are necessarily broad, and thus require tailoring to best fit MBS contexts. In addition, future qualitative research is needed to provide much needed insight into patients’ lived experienced related to the intersection of trauma with MBS. Such work is important as quantitative research often, and unfortunately, loses the individualized experience of a given patient that can be critically important to the delivery of TIC. Qualitative research could complement quantitative studies by providing deeper contextual understanding of how the pre- and postoperative process are experienced by individuals who have a history of trauma.

While the last two decades have witnessed tremendous growth in research on the psychosocial issues in bariatric surgery, studies on the ACEs-MBS relationship have lagged. Future research should examine how social determinants of health and ACEs influence the development of class III obesity as well as MBS outcomes. While MBS remains the most efficacious tool to treat obesity, patients have to use that tool in an environment that is often far from supportive with respect to promoting health behaviors consistent with positive postoperative outcomes. That same environment also may be one in which trauma occurred or its echoes resonate regularly. While public health professionals will likely need to lead the charge in identifying health promoting strategies related to social determinants of health, members of the MBS team can prepare to provide compassion trauma-informed care to that large percentage of patients who present for surgery with a history of trauma.
